# HAPmini: 2D haptic feedback generation using single actuator device

**DOI:** 10.1371/journal.pone.0285002

**Published:** 2023-04-26

**Authors:** Hwan Kim, Kyung Hoon Hyun

**Affiliations:** Department of Interior Architecture Design, Hanyang University, Seoul, Republic of Korea; Virginia Tech: Virginia Polytechnic Institute and State University, UNITED STATES

## Abstract

This study aims to explore a feasible form of a haptic device for common users. We propose HAPmini, a novel graspable haptic device that enhances the user’s touch interaction. To achieve this enhancement, the HAPmini is designed with low mechanical complexity, few actuators, and a simple structure, while still providing force and tactile feedback to users. Despite having a single solenoid-magnet actuator and a simple structure, the HAPmini can provide haptic feedback corresponding to a user’s 2-dimensional touch interaction. Based on the force and tactile feedback, *the hardware magnetic snap function* and *virtual texture* were developed. *The hardware magnetic snap function* helped users perform pointing tasks by applying an external force to their fingers to enhance their touch interaction performance. *The virtual texture* simulated the surface texture of a specific material through vibration and delivered a haptic sensation to users. In this study, five *virtual textures* (i.e., reproductions of the textures of paper, jean, wood, sandpaper, and cardboard) were designed for HAPmini. Both HAPmini functions were tested in three experiments. First, a comparative experiment was conducted, and it was confirmed that *the hardware magnetic snap function* could increase the performance of pointing tasks to the same extent as *the software magnetic snap function* could, which is commonly used in graphical tools. Second, ABX and matching tests were conducted to determine whether HAPmini could generate *the five virtual textures*, which were designed differently and sufficiently well for the participants to be distinguished from each other. The correctness rates of the ABX and the matching tests were 97.3% and 93.3%, respectively. The results confirmed that the participants could distinguish *the virtual textures* generated using HAPmini. The experiments indicate that HAPmini enhances the usability of touch interaction (*hardware magnetic snap function*) and also provides additional texture information that was previously unavailable on the touchscreen (*virtual texture*).

## 1. Introduction

Early haptic devices, developed a few decades ago, were extremely large and intricate for daily use. After the first haptic device was proposed by Rosenberg in 1992 to enhance usability while remotely controlling surgical robots [1], researchers in haptics and human-computer interaction (HCI) have explored its feasible form for end-user touch interaction [2–7]. Various types of haptic devices have been proposed, ranging from heavy and grounded devices with robot arms [1,8] to exoskeletons [9–11], braille-type tactile displays [12], as well as light and embedded devices that use vibrations [2,13–15] or electroadhesion [16]. These devices reproduce rich haptic sensations, such as repulsive forces [8,17–19] or frictional forces [15,20], while touching an object, or its elasticity [15,17,21] or texture [22–26] in a manner. Henceforth, researchers have strived toward enhancing devices to make them more suitable for common users to incorporate into their daily lives. Compared to the researchers’ state-of-the-art devices, the haptic sensation of some commercialized devices, such as the controllers of PlayStation or Meta Quest 2, is closer to a buzzing sensation rather than the rich haptic sensation [27]. However, commercially available devices have a significantly simpler mechanical structure than those from the literature. To reproduce a more plausible haptic sensation beyond the simple buzzing sensation while maintaining a relatively simple mechanical structure, haptic devices have been developed to operate in multiple degrees of freedom (DOFs) or multiple dimensions; they are required to comprise multiple actuators and sophisticated mechanical structures. HapCube [15] is a small tactile feedback device (24 mm × 24 mm × 20 mm) that generates multidimensional vibrotactile feedback but has a complicated x-y stage structure and multiple actuators. Schorr and Okamura [7] developed a finger-worn tactile device that delivers 3-DOF tactile feedback to a user’s finger pad, and Mintchev et al. [3] designed a thin joystick (3 mm thickness) with an origami structure that creates 3-DOF force feedback. They utilized sophisticated DELTA robot structures and three motors for multiDOF feedback. To provide a highly articulated haptic experience, a haptic device typically requires a combination of multiple actuators that synthesize movements into multidimensional haptic feedback. However, a complex design with numerous components may pose a challenge for consumer products [2]. Therefore, reducing the mechanical complexity of haptic devices may be beneficial for common users by enabling a more streamlined and accessible haptic experience.

**Fig 1:**
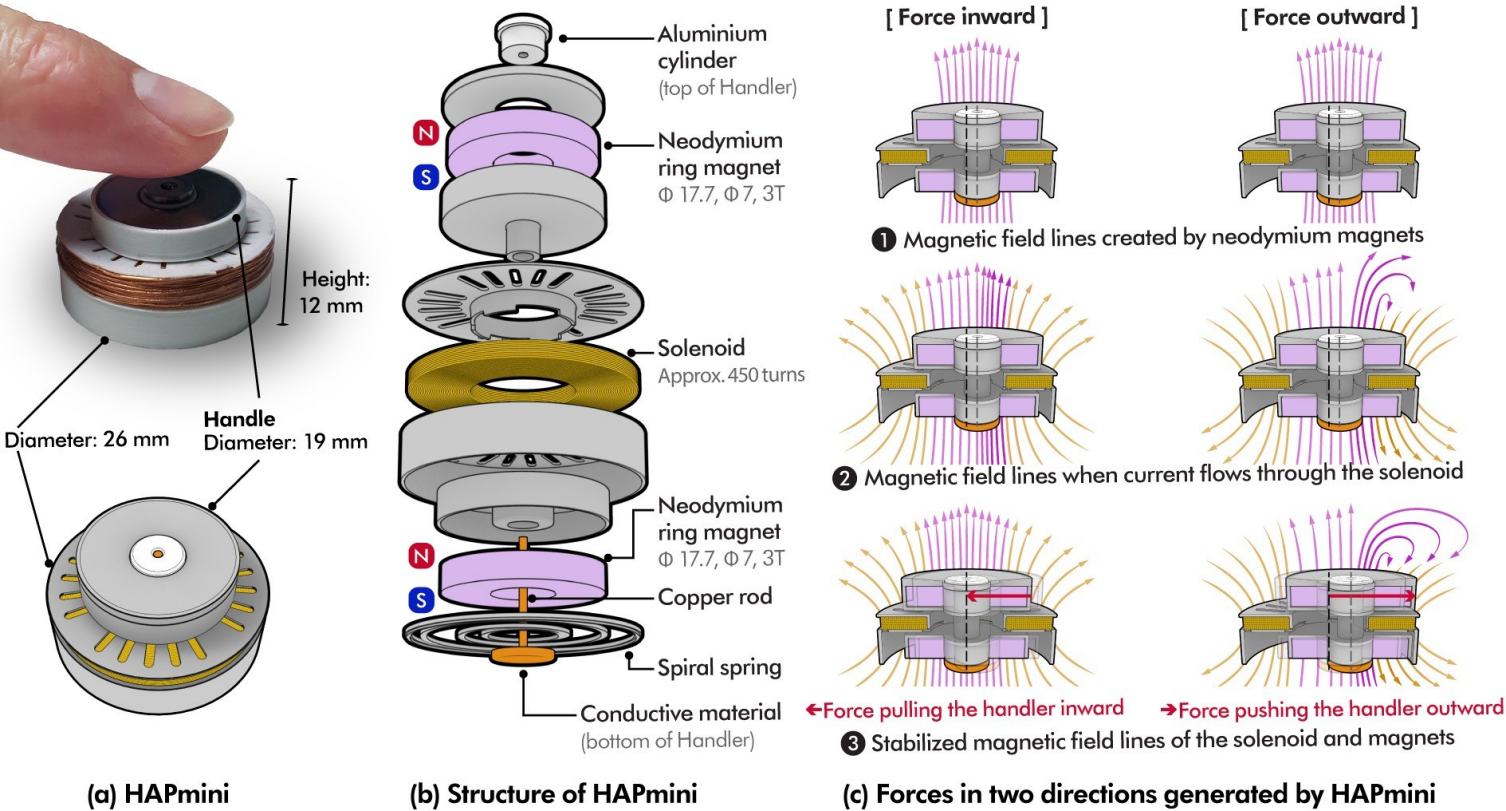


Thus, this study aims to explore a method to provide haptic feedback for supporting the user’s touch interactions with lower complexity, fewer actuators, and a simple mechanical structure; as such, we propose HAPmini as a feasible alternative to exploration (Fig 1). HAPmini is a joystick-type input and haptic device that responds to a user’s two-dimensional touch interaction through 1-DOF actuation. HAPmini provides two types of haptic feedback (force and tactile) in response to a user’s two-dimensional manipulation on a touchscreen. It consists of a solenoid-magnet actuator and spiral spring. The actuator consists of a solenoid and a permanent magnet in coaxial alignment, which enables the user to manipulate the HAPmini in a tangential two-dimensional plane. At this point, if the polarity of the voltage applied to the solenoid changes, a pushing or pulling force can be applied in the direction of user operation. This enables instantaneous physically capture of a user’s operation via force feedback and the creation of various tactile sensations via vibrotactile feedback. We implemented a magnetic snap function (*hardware magnetic snap function*) that can help the user’s pointing task using force feedback and a function that simulates various textures on the touchscreen using this vibrotactile feedback (*virtual texture*).

First, *the hardware magnetic snap function* is a physical implementation of *the software magnetic snap function* through hardware (i.e., HAPmini). *Software magnetic snap function*, which is commonly used in tools, such as Adobe Illustrator and Microsoft PowerPoint, enables users to select a specific object more easily or to accurately move its position by briefly holding a cursor over the object [28]. To validate whether *the hardware magnetic snap function* can help with the user’s pointing task, we conducted a comparative experiment and confirmed that *the hardware magnetic snap function* was as helpful as *the software magnetic snap function*. Second, five different *virtual textures* (reproductions of the textures of paper, wood, jean, sandpaper, and cardboard) for HAPmini were designed and tested. ABX and matching tests were conducted to confirm whether HAPmini can provide distinctive and realistic *virtual textures* to users. The results of the ABX test indicated that *the five virtual textures* generated by the HAPmini could be distinguished based on the human tactile sense. The experiment aimed to ascertain the distinguishability of each pair of *virtual textures*; however, it did not demonstrate their fidelity to *the original textures*. A test was conducted to match each *virtual texture* with the corresponding *original texture*. In this test, the participants were blindfolded and given five *virtual textures* and then five *original textures* openly. The results indicated that they successfully matched the corresponding textures.

The HAPmini is a haptic device designed to enhance touch interaction by providing users with *the hardware magnetic snap function* and *virtual textures* using a single solenoid-magnet actuator. Although previous studies proposed similar haptic devices to enhance touch interaction, they relied on multiple actuators and required precise mechanical structures to combine their actuations [3,7,14,15]. By contrast, this study aims to develop a haptic device that can provide rich haptic sensations with few actuators and simple structures, similar to commercially available devices. Specifically, we propose a 1-DOF actuation haptic device that corresponds to a user’s two-dimensional touch interaction. A reduction in the mechanical complexity of haptic devices will make them more affordable for common users. The experiments indicate that HAPmini enhances the usability of touch interaction (*hardware magnetic snap function*) and provides additional texture information that was previously unavailable on the touchscreen (*virtual texture*). Using simple structures and a few components, we aim to make haptic devices more accessible to general users.

## 2. Related works

### 2.1. Auxiliary devices to assist touch interaction

The direct manipulation of GUI elements on a touchscreen with fingers is an intuitive interaction method. However, the currently available interaction methods with fingers on the touchscreen do not completely utilize the high dexterity of the human hands compared to the mouse and keyboards (i.e., PUIs). In this respect, researchers have developed methods to extend the interaction space of touchscreens by adding small add-on devices that touchscreens can recognize. For example, Jordà [29] installed a projector and camera under a tabletop and developed a table system that displayed images and recognized the user’s touch and objects. A QR code printed on an object allows the reactive system to identify the object and to track its position and orientation. Additionally, methods for recognizing the position and orientation of add-on devices using capacitive, light, and Hall sensors have been proposed [30–34]. Users can use these objects as joysticks, sliders, dials, and buttons on touch screens. Recently, a method for recognizing object deformation (press, squeeze, or bend) as an input method in a game [35] and a method for actuating an object using an array of electromagnets [36] have been proposed. Assistive devices for touchscreens have been developed to improve their usability by allowing tangible interaction among users. Users can obtain new input methods for touchscreens by directly manipulating objects by using their hands. However, the aforementioned literature on assistive devices has mainly focused on expanding the input channel, and has been limited to expanding haptic feedback modes. The HAPmini proposed in this study is an auxiliary input device for touchscreens and a haptic device that generates tactile information.

### 2.2. Haptic constraints to increase usability

When drawing a straight line with a pen, a ruler helps in drawing the line more quickly and accurately by limiting the pen’s free movement. In this case, the ruler acts as a physical constraint, promoting usability by restricting user behavior. After the GUI became the standard with the introduction of the mouse and cursor, HCI researchers explored visual constraints to increase the usability of user interactions [37–40]. For example, *the software snap function* aids users in pointing at exact positions, and dimmed buttons help users understand that a button is not clickable. Li et al. [41] utilized virtual resistance and visual deformation to increase stiffness perception while simulating soft object surfaces as an alternative to vibrotactile feedback on consumer tablet PCs. In addition, 3D modeling tools utilize methods to move and rotate objects and viewports along a single orthogonal axis for convenient manipulation. According to Fitts’ law, placing frequently used buttons (e.g., the start button in Windows OS) on the outermost part of a display is another way to utilize visual constraints to increase usability [42]. In this manner, constraints are used to enable users to perform interactions more accurately and easily. Numerous researchers in HCI and haptics fields have proposed methods for implementing haptic constraints that mimic physical or visual constraints by providing haptic feedback [17,37,42]. Chan et al. [18] demonstrated that haptic feedback when touching a virtual touchscreen mid-air can improve user performance, whereas Rosenberg [1] proposed a method in which force feedback assists robotic surgery by limiting entry into hazardous areas. Haptic constraints enhance users’ task performance by restricting their movements.

### 2.3. Haptic device for touch interaction

Haptic feedback is known to improve haptic interfaces [43]. Recently, haptic feedback has been used in virtual reality (VR) and augmented reality (AR). Wang and MacKenzie [37] used force feedback as a haptic constraint when a user manually manipulated a virtual object in VR and AR environments. The haptic device creates haptic constraints by intervening in the user’s manipulation, rather than simply reproducing tactile information (e.g., the feel of texture). For example, the master interface used in robotic surgery provides haptic constraints (force feedback) during the surgery and physically prevents the surgical tool from approaching dangerous areas [1,44]. Wu et al. [39] proposed revising the pen’s position on a virtual plane with a PHANToM haptic device to help the users write with a pen in air. In addition to physically blocking a user’s hand movement, a method for improving the performance of touch interaction by providing appropriate haptic guidance for the user’s operation has been proposed. Zoran et al. [45] developed a handheld drill for sculpting that turned off its motor when the drill pointed to a predetermined area. The drill prevents the user from mistakenly sculpting an important volume of material. Additionally, Kim et al. [17] found that the performance of click and drag operations was improved by providing only haptic feedback to the virtual buttons on touchscreens. Recently, as the VR market has matured and the number of VR users has increased, research on developing haptic devices for commercialization and common users has been proposed [2–4]. The goal of researchers is to develop a haptic devices with low mechanical complexity. Mintchev et al. [3] developed a thin haptic joystick that could be easily installed anywhere using an origami structure. Lee et al. [2] proposed a VR controller that provides tactile feedback with a rigid body using load cells and vibration motors. In addition, many researchers have proposed haptic devices for use in people’s daily lives [4–6].

Despite the aforementioned state-of-the-art literature, the quality of haptic feedback and the mechanical complexity of haptic devices usually have a trade-off relationship, and a haptic device with low mechanical complexity that can simultaneously provide plausible force and tactile feedback has not been proposed. Therefore, this study aims to develop an auxiliary haptic device that provides force feedback to increase the usability of touch interactions and tactile feedback to reproduce textures.

### 2.4. Texture rendering technique through vibrotactile feedback

Previous studies have proposed texture-rendering techniques based on their theoretical rationale. Asano et al. [23,25] proposed a method for reducing the roughness of the surface by making the surface feel slippery with a high-frequency vibration of 250 Hz. In addition, a method for increasing the perceived roughness of the surface by generating vibrations according to the moving distance of the finger was proposed [23,25]. However, this method cannot reproduce the unique textures of objects (e.g., wood grains and sandpaper roughness). Vijaya et al. [26] proposed a process for obtaining *virtual textures* from real materials. According to them, changes in acceleration obtained by scratching the surface of a real material with a probe equipped with an accelerometer reflect the texture of the surface. Consequently, *a virtual texture* can be generated using acceleration changes and a vibration motor. Romano et al. [24] and Kuchenbecker et al. [22] proposed a more realistic texture reproduction method by varying the vibration pattern according to the user’s touching speed, acceleration, and the normal force on the surface. However, the best way to generate *the virtual texture* has not yet been confirmed, and researchers are continuously improving new techniques. In this study, the method proposed by Vijaya et al. [26] for generating texture feedback by collecting acceleration data from real materials was modified for HAPmini’s indirect manipulation method.

## 3. HAPmini

### 3.1. Hardware design

The HAPmini is a device with a solenoid magnet actuator. Its dimensions were approximately 26 mm × 26 mm (diameter) × 12 mm (height), and it was used on a touch screen. The top and bottom of the handle were connected with conductive materials (a copper rod and an aluminum cylinder; copper and aluminum are paramagnetic and do not react to the magnetic force) to allow the touch screen to recognize the joystick of the HAPmini (Fig 1b). A spiral spring connected to the bottom of the handle provides a restoring force that returns the joystick to its center position when it is moved. The moving range of the HAP mini-joystick was approximately ± 6 mm in the two-dimensional direction. The HAPmini structure was fabricated using a 3D printer (Ultimaker S5 Pro).

Solenoid-magnet actuators respond more quickly to changes in electrical signals than do other actuators (e.g., DC motors). Therefore, it is advantageous not only for force feedback, but also for generating vibrotactile feedback that requires a rapid response speed. However, in previous studies, solenoids and magnets were aligned in a straight line; such that each actuator could only generate one-dimensional linear motion. Therefore, to correspond to multidimensional operations, such as joystick operations, it was necessary to combine multiple actuators [14,15,20]. We attempted to find a way to respond to the manipulation movement in two dimensions while using as few actuators as possible and designed the HAPmini using a coaxial alignment. The HAPmini can provide haptic feedback for the movement of the joystick in two dimensions using only one solenoid-magnet actuator.

The force and tactile feedback of HAPmini were generated by pulling the handle inward and pushing it outward. This force is generated by the electromagnetic force of the coaxially aligned neodymium magnet and solenoid. At the top and bottom of the handle, the neodymium magnets were positioned in the same polarity direction, and the solenoid was positioned between them. The direction of this force (pulling or pushing) and its strength can be controlled by the strength and polarity of the current flowing through the solenoid. When a positive electric current is applied to the solenoid (Fig 1c), an electromagnetic force is applied toward the center of the HAPmini, where the magnetic flux density becomes constant (force inward). When a negative electric current was applied to the solenoid, an electromagnetic force was applied to the handle away from the center of the HAPmini (outward force). HAPmini lacks the ability to direct electromagnetic force in two dimensions; however, it is possible to guide two-dimensional finger movements.

**Fig 2:**
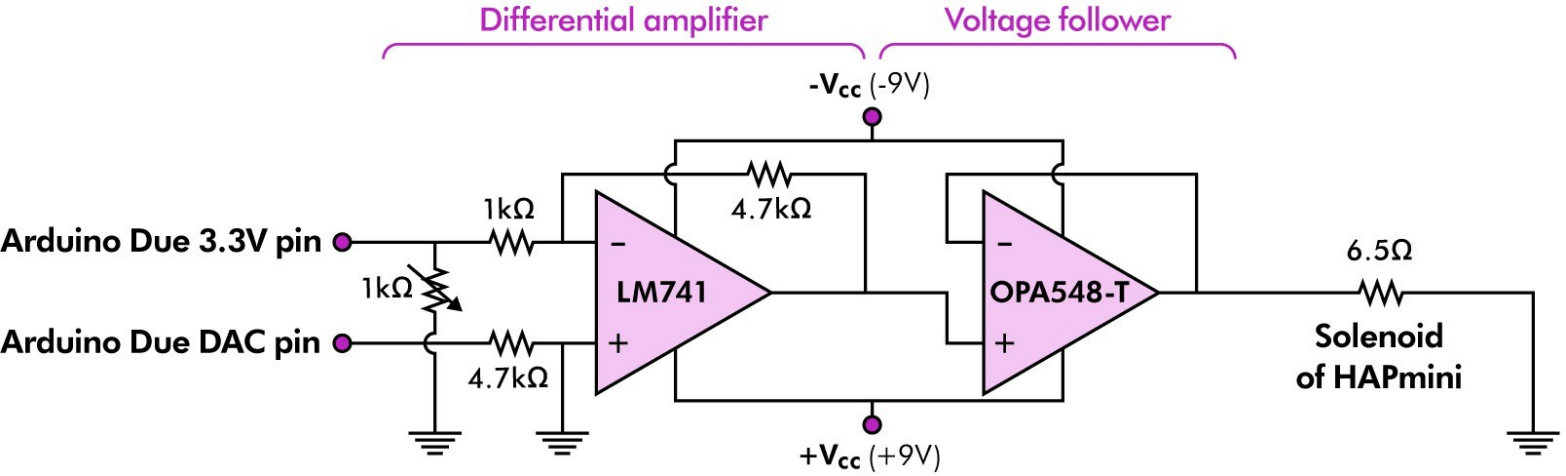


The magnetic force of the solenoid was controlled using an Arduino Due and two types of operational amplifiers (Op-Amps) (Fig 2). The differential amplifier of LM741 amplifies the analogous voltage signals (0–3.3V) of Arduino Due’s digital-to-analog converter (DAC) channels to a dual-polarity voltage signal (± 6.7V), allowing the polarity of the solenoid to be adjusted as desired. The voltage follower’s OPA548-T can handle up to 5A (continuous output current:3A; peak output current:5A) and supply sufficient current to the solenoid. The measured resistance of the solenoid was approximately 6.5 Ω, indicating that a maximum current of approximately 1.0 A can be supplied to the solenoid.

### 3.2. Feedback design

HAPmini can provide both force and tactile feedback. We implemented *the hardware magnetic snap function* and *virtual textures*. The force feedback of the HAPmini was designed for *the hardware magnetic snap function* to assist the user’s pointing task with physical force. The *virtual textures* were designed to render an object’s surface texture using the vibrotactile feedback of HAPmini. This section describes the designs: 1) the *hardware magnetic snap function* and 2) *virtual textures*.

#### 3.2.1 Hardware magnetic snap function design

**Fig 3:**
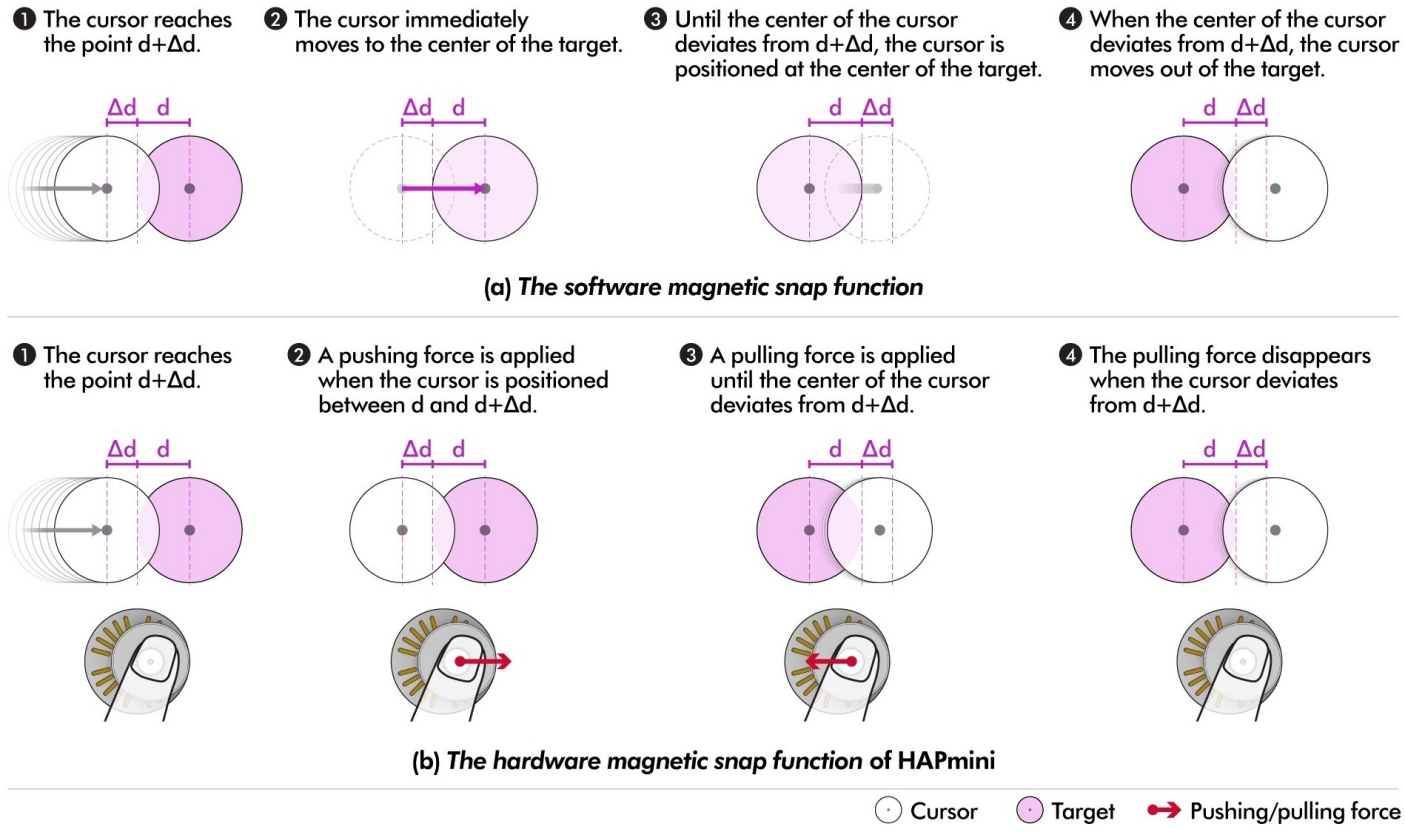


*The software magnetic snap function* helps the user’s pointing task by making a cursor stick to the area of the button when the cursor approaches or passes over the button. For example, when moving a shape in MS PowerPoint, the shape sticks to other shapes, such as a magnet. Various methods to implement the software magnetic snap function have been proposed [28,46], and the most popular method is to set the selectable area (Δd in Fig 3a) slightly wider than the visible target area (d in Fig 3a). When the cursor reaches point d+Δd, the cursor position is moved to the center of the target such that it can be positioned within the target area. When the cursor moves outward, the cursor position is moved out of the target when it reaches d+Δd. This method is also often used in design support tools like Adobe Illustrator and Rhinoceros 3D to help a user’s pointing and dragging tasks.

The *hardware magnetic snap function* was designed under the same conditions as the software magnetic snap function (Fig 3b). However, in contrast to the software magnetic snap that immediately moves the cursor to the center of the target, the hardware magnetic snap applies a pushing force to the joystick to move the cursor to the target more quickly. The pushing force was maintained until the cursor was between d+Δd and d. When the cursor was within the target range (0 − d+Δd), a pulling force was continuously applied to the joystick. This pulling force makes the user to apply more force when the cursor attempts to exit the target, thereby keeping the cursor position within the target range. The Δd value of 35px (based on a 3920 × 2160 px resolution display) was set, consistent with Adobe Illustrator’s similar value.

In addition, the average of the maximum pushing force and the average of the maximum pulling force of the HAPmini, measured ten times using a tension gauge (Roland Tension Gauge 500 g), were approximately 3.26 N for pushing and 2.73 N for pulling. This force corresponds to objects weighing approximately 332 and 278 g, respectively, which exceeds the weight of the iPhone 14 Pro Max (approximately 240 g).

#### 3.2.2 Virtual texture design

**Fig 4:**
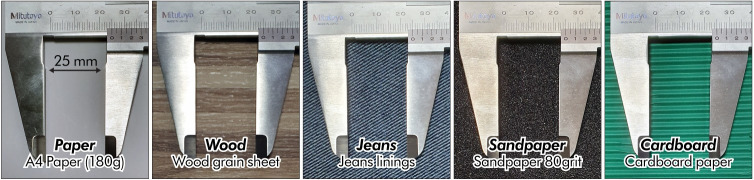


To provide more realistic texture feedback, the texture was designed from the acceleration data obtained by scraping the actual materials. We collected acceleration data from five materials and obtained five texture signals through signal processing. To determine whether HAPmini can generate various texture feedbacks, we selected five materials (paper, wood, jean, sandpaper, and cardboard; Fig 4). Paper is one of the most common materials, and its roughness is significantly lower than that of the other materials. Wood and jeans have unique grains, but the texture of the jean is smoother. We selected 80-grit sandpaper following the CAMI system, and its particle size was approximately 200 μm. The particle size was much smaller than the spacing between the grains of wood or jeans; however, the texture of the sandpaper was considerably rougher because of its hard surface. Finally, cardboard has a distinctive bumpiness with significantly larger ridges than the other materials.

**Fig 5:**
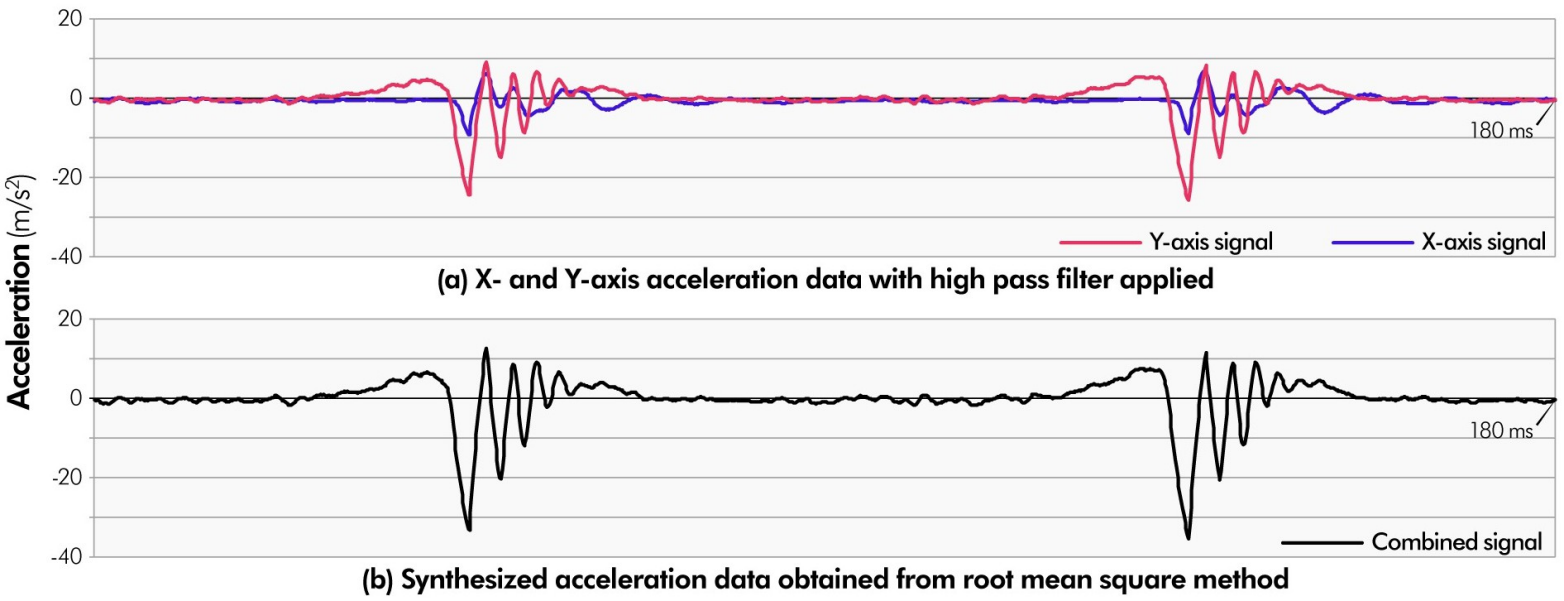


The five materials were attached to a rigid acrylic plate to collect acceleration data. The surface of each material was scraped in one direction using a thin cutter knife equipped with a 3-axis accelerometer (ADXL335). The acceleration changes were recorded for approximately 10 s per surface. The acceleration data of the X- and Y-axes were recorded at 6666.7 Hz (recording time interval:150 μs) using the Arduino Uno and Ethernet shield 2. To eliminate the unnecessary effects caused by changes in the angle, direction, and speed of the knife, we applied a 10 Hz high pass filter to the acceleration data. Fig 5a shows the results of applying the high-pass filter to the acceleration data of the cardboard. The human tactile system is insensitive to orientation [47]; Romano et al. [24] and Kuchenbecker et al. [22] proposed methods for synthesizing three-dimensional acceleration data by considering variations in finger speed, angle, and pressure. These methods are designed for situations in which a user’s finger moves directly on a surface (i.e., a touchpad or touchscreen). However, the HAPmini design assumes the form of a joystick, an indirect manipulation method that moves the cursor. Therefore, they could not reflect the angle and pressure of the cursor, and their methods [22,24] did not apply to HAPmini. Only changes in speed can be reflected. To reflect only the speed of the cursor, the acceleration data along the X- and Y-axes were synthesized into a single signal following Vijaya’s method [26].

Based on the data showing the dominant acceleration change among the acceleration data of the X- and Y-axes, the power of the acceleration data of each axes was obtained through the root mean square. Their sum is then multiplied by the acceleration data of the Y-axis to obtain a single combined acceleration data ([(RMSx + RMSy) / RMSy]), where RMSx and RMSy are the root mean square values of the X- and Y-axis signals (see Fig 5b).

**Fig 6:**
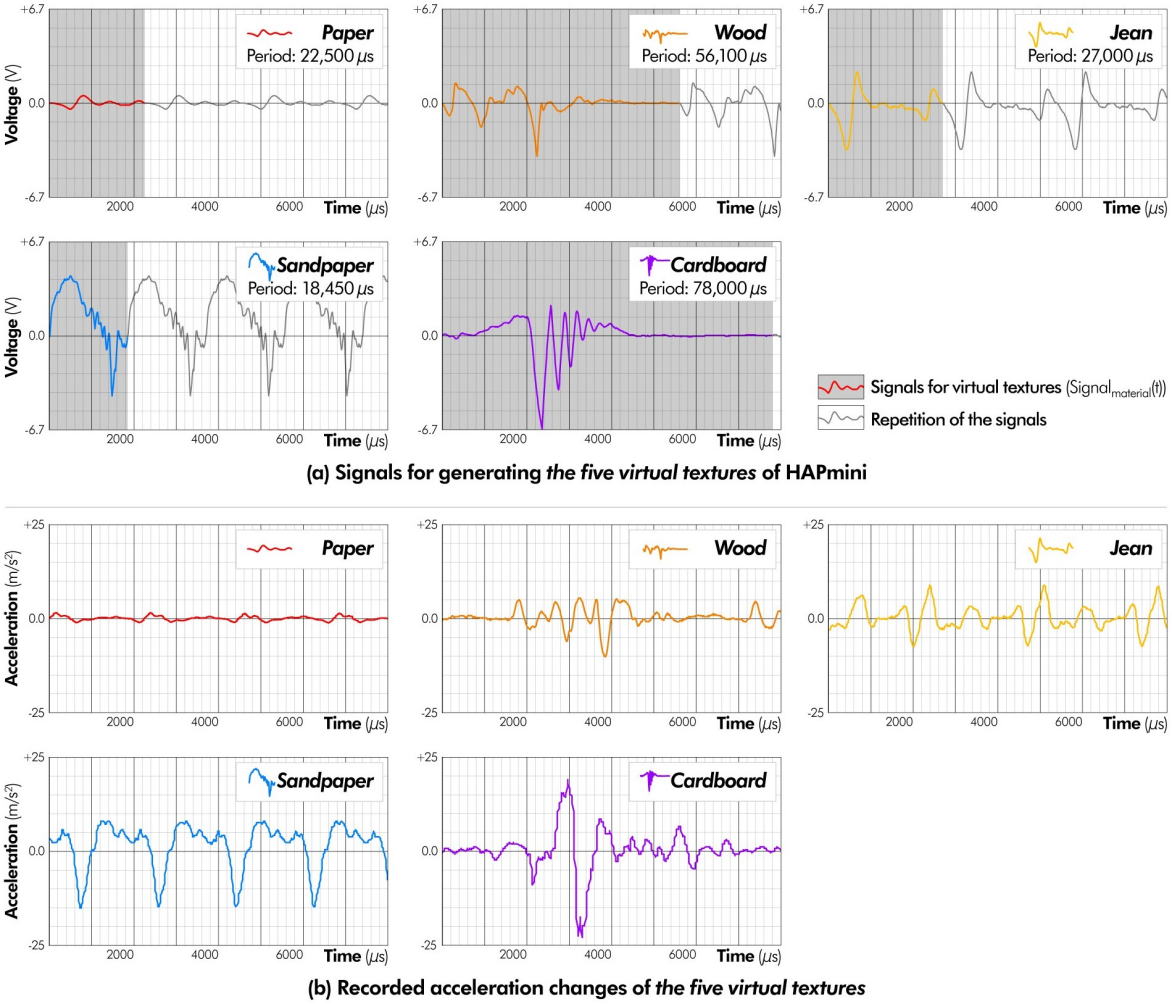


All combined signals consisted of repetitions of small vibration patterns, and these repeated cycles were used to calculate the signals of the *virtual textures* (Signal_material_(t)). Fig 6a shows the repeated cycles of five materials (Acc_material_(t), where t is time (μs)), and the signals of the *virtual textures* are determined by the following formula, considering the speed of the cursor (S):

$$Signal_{material}\left(t\right)=\;6.7\;V\;\times \;Acc_{material}\left(wt\right)$$

$$\left(w\;\propto \;\;S,\;\;w\;\;becomes\;\;1.0\;\;when\;S\;is\;15\;mm/s,\;material\;is\;one\;of\;\left\{Paper,\;\;Wood,\;Jean,\;Sandpaper,\;Cardboard\right\}\right)$$

Before conducting the user studies (ABX and matching tests), we conducted a measurement experiment to confirm that HAPmini generate the five *virtual textures* properly. The vibrations of the *virtual textures* were measured using the accelerometer for approximately 10 s. The value of w was set to 1.0. Fig 6b shows the measurement results after the high-pass filter and X- and Y-axis syntheses. These results show that HAPmini effectively generated five *virtual textures* according to the designed signals.

## 4. User experiments

Three user experiments were conducted to confirm whether HAPmini enhanced the user’s touch interactions. The first experiment was conducted to determine whether the *hardware magnetic snap function* (force feedback) of the HAPmini could improve the performance of a user’s pointing task. The second and third experiments were conducted to determine whether users could distinguish and perceive the five *virtual textures* generated by HAPmini. Specifically, a second experiment, the ABX test was conducted to determine whether users could distinguish between the five types of *virtual textures*. The third experiment was a matching test to ascertain the participants’ ability to infer *the original texture* from the reproduced *virtual texture*. These experiments complemented each other to assess participants’ perception of haptic feedback from the device [15,21]. The results of the ABX test demonstrated that HAPmini can deliver *virtual textures* that are distinguishable from each other at a minimum. That is, if *the original textures* are different, the *virtual textures* are perceived as distinct. The results of the matching test show how well participants can recognize *the original textures* from which the virtual textures come and how well HAPmini expresses the characteristics of the original textures. All the experiments were approved by the Hanyang University Institutional Review Board (No: HYUIRB-202206-005-1). All participants were recruited through offline flyers at Hanyang University and received approximately 4 USD as a reward for participating in the experiment.

### 4.1. Experiment 1: Pointing task

#### 4.1.1 Experiment design

The pointing task experiment was performed under the following six device conditions: touchscreen-no snap (**TN**), touchscreen-software snap (**TS**), HAPmini-no snap (**NN**), Hapmini-software snap (**NS**), HAPmini-hardware snap (**HN**), and HAPmini-hardware and software snap (**HS**) (Table 1). Participants performed the pointing task in the **TS** and **TN** conditions using a software joystick implemented as a GUI on the touchscreen. A software magnetic snap function was provided in the **TS**, although it was not provided in the **TN**. In the **HS**, **HN**, **NS**, and **NN** conditions, participants performed the pointing task using HAPmini on the touchscreen. For **HS**, both *hardware and software magnetic snap functions* were utilized. Only the *hardware magnetic snap function* was used under the **HN** condition. Only the *software magnetic snap function* was provided for the **NS** condition, and no snap function was provided for the **NN** condition.

**Table 1:** Device conditions of experiment 1.

Device condition	HS	HN	NS	NN	TS	TN
Input method	Hardware joystick				Software joystick	
Hardware magnetic snap	O	O	X	X	X	X
Software magnetic snap	O	X	O	X	O	X

**Fig 7:**
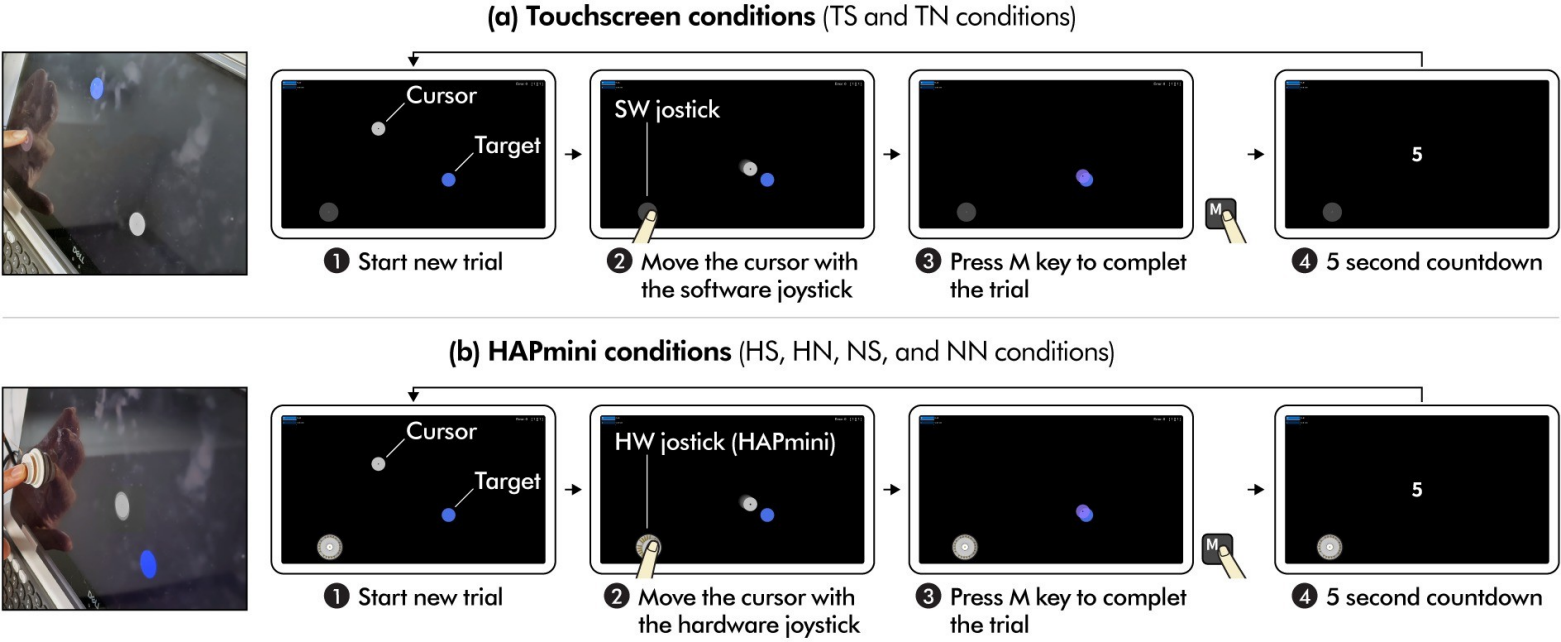


The participants moved the cursor (white circle in Fig 7a) into the target (blue circle in Fig 7a) using the software or the hardware joystick (HAPmini) and then pressed the ‘M’ key when the center of the cursor enters the area of the target to complete the task. The diameters of the cursor and the target were approximately 20 mm. The task does not end even if the ‘M’ key is pressed outside the target and the participant must perform the pointing task until it succeeds. The duration of the task completion was measured. The initial distance between the cursor and the target was 130 mm from the center of the screen, and their positions were randomly defined. When the center of the cursor was positioned above the target, the color of the cursor changed to bright violet. If the ‘M’ key was pressed, the task was completed, a 5-second countdown was provided, and then the next task was provided. The value of Δd was set as 35 px (approximately 5 mm) for the *hardware and software magnetic snap functions*.

Twelve participants were recruited for the experiment (Age_mean_ = 27.67; Age_SD_ = 4.46; five females and seven males). The number of participants was determined according to the typical participant size (10–20) in the HCI field (12 participants in Kuchenbecker et al. [22]; 10 participants in Kim et al. [15]; 8 participants in Asano et al. [23]). The participants performed the pointing task under six device conditions, and the order of the conditions was randomly determined to minimize the order effect. Each device condition consisted of five sessions, and the participants repeatedly performed the pointing task ten times in each session. In other words, the participants performed the pointing task 50 times and then repeated the pointing task in the remaining device conditions. After completing all the tasks, the NASA TLX [48] survey was conducted to evaluate the perceived workload according to the conditions. Each participant required an average of approximately 1 h and 30 min to complete the experiment under all device conditions. A touchscreen laptop (Precision 5530 2-in-1, Dell Inc.) was used to conduct this experiment.

#### 4.1.2 Experimental results

**Fig 8:**
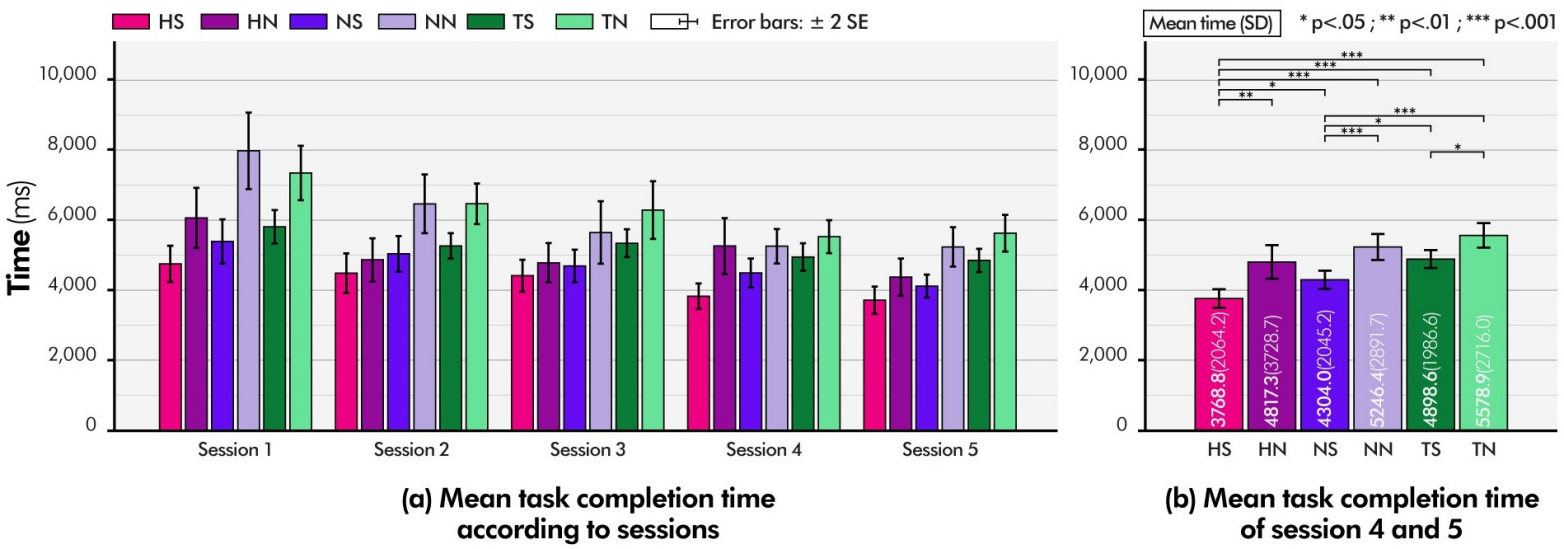


The changes in the mean task completion time according to sessions are shown in Fig 8a. The participants became familiar with the pointing task as the session progressed and their task performance improved. However, from the fourth session onward, no statistically significant difference was found between the fourth and fifth sessions (Bonferroni pairwise comparison, p = 0.61), and the learning effect disappeared from the fourth session. To exclude bias caused by the learning effect, we combined the task performances of the fourth and fifth sessions for comparison. Fig 8b shows the mean task completion times of the fourth and fifth sessions according to the device conditions. In general, the experimental results showed that the subjects could complete the pointing task slightly faster under the conditions using HAPmini (**HS**, **HN**, **NS**, and **NN**) than under the conditions using the software joystick of the touchscreen (**TN** and **TS**). In addition, the participants showed that they could complete the pointing task faster under conditions in which *the software and hardware snap functions* were provided (**HS**, **HN**, **NS**, and **TS**) than under the other two conditions without the snap function (**TN** and **NN**).

Both the snap functions significantly reduced the completion time of the pointing task. The **TN** condition required the most time to complete the task (M = 5578.9 ms; SD = 2716.0 ms). However, the mean task completion time of the **TS** condition was 4898.6 ms (SD = 1989.6), which was significantly faster than the **TN** condition (Bonferroni pairwise comparison, p < 0.05). The effect of *the software snap function* was also observed while using a hardware joystick. **NN** and **NS** conditions required an average of 5246.4 ms (SD = 2791.7) and 4304.0 ms (SD = 2045.2), and the **NS** condition was significantly faster than **the NN** condition (Bonferroni pairwise comparison, p < 0.01). *The software snap function* assisted the participants in performing pointing tasks faster on both of the software and hardware joysticks. In the **HN** condition where only *the hardware snap function* was provided, the mean task completion time was 4817.3 ms (SD = 3728.7), which was slightly longer than the duration of the **NS** condition, although no statistically significant difference was found (Bonferroni pairwise comparison, p = 0.979). The **HS** condition, for which both of *hardware and software snap functions* were provided together, had the fastest task completion time (3768.8 ms; SD = 2064.2), which was significantly faster than all other five device conditions (Bonferroni pairwise comparison, all ps < 0.05). The data demonstrate that *the hardware magnetic snap function* can improve user performance in pointing tasks to the same degree as *the software magnetic snap function* and that combining the two further boosts performance. HAPmini’s *hardware magnetic snap function* applies physical force to the user’s finger, helping them perform their pointing tasks stably and quickly.

**Fig 9:**
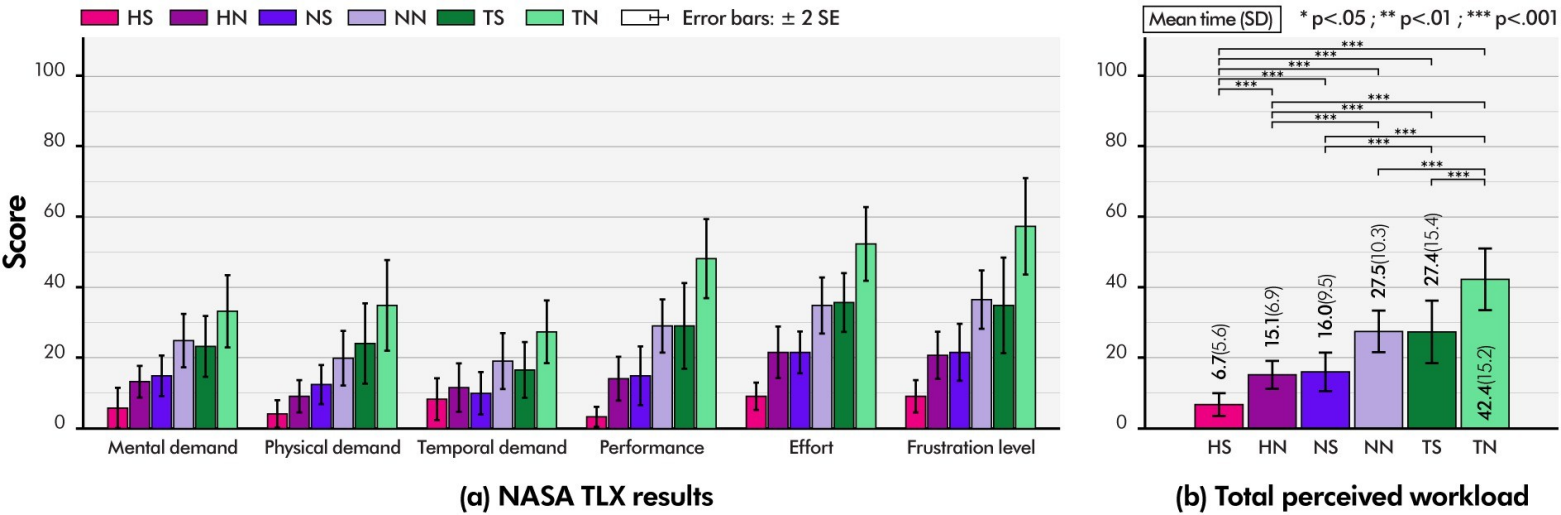


The NASA TLX results showed a trend similar to that of task completion time (Fig 9). Similarly, the perceived workload in the **HS** condition was the lowest (Bonferroni pairwise comparison, all ps < 0.001; Fig 9b), and the perceived workload in the **TN** condition was the highest (Bonferroni pairwise comparison, all ps < 0.001; Fig 9b). The perceived workloads in the **NN** and **TS** conditions were similar (Bonferroni pairwise comparison, p = 1.0). This confirms that the provision of auxiliary hardware input devices, such as HAPmini (although *the hardware magnetic snap function* is not provided), can assist touch interaction as much as *the software snap functions* in touchscreens. The perceived workloads in the **HN** and **NS** conditions were similar with no significant differences (Bonferroni pairwise comparison, p = 1.0). The NASA TLX results also indicated that *the hardware and software magnetic snap functions* enabled participants to complete the pointing task with a low workload.

Based on the results of this experiment, it was determined that *the hardware magnetic snap function* is as effective in assisting touch interactions as *the software magnetic snap function*. Furthermore, when used in conjunction with HAPmini’s hardware and the software snap functions, the performance of the user’s pointing task can be significantly improved. Whereas *the software magnetic snap function* indirectly affects the user’s fingers by intervening in the movement of graphical elements within the display, *the hardware magnetic snap function* directly enhances touch interactions by interfering with the user’s finger movements, enabling quicker and more stable performance. This suggests that the force feedback of the HAPmini can influence the user’s fingertip movement to mimic the constraints of physical objects in the real world, similar to using a ruler to draw straight lines more accurately and quickly.

### 4.2. Experiment 2–1: ABX test

#### 4.2.1 Experiment design

**Fig 10:**
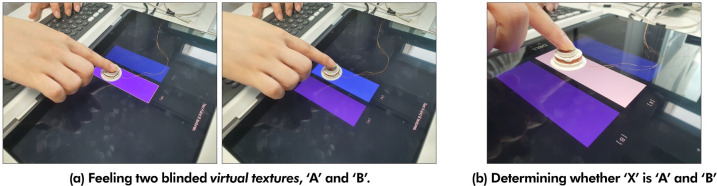


In Experiment 2, the ABX test was conducted to determine whether participants could distinguish HAPmini’s *virtual textures (****Paper***_***v***_, ***Wood***_***v***_, ***Jean***_***v***_, ***Sandpaper***_***v***_, *and* ***Cardboard***_***v***_*)*. This test shows how well HAPmini reproduces the different nuances of the original textures (***Paper***_***p***_, ***Wood***_***p***_, ***Jean***_***v***_, ***Sandpaper***_***p***_, *and* ***Cardboard***_***p***_). The ABX test is used to distinguish whether one of the two stimuli is A or B when one (X) of the two stimuli is provided to the participant randomly and blindly. To perform the ABX test, five *virtual textures* of HAPmini were divided into 10 pairs. For example, when ***Wood***_***v***_ and ***Jean***_***v***_ were provided to participants as A and B, each participant had sufficient time to feel the haptic sensations of ***Wood***_***v***_ and ***Jean***_***v***_ created by the HAPmini (Fig 10a). One of the two feedbacks was then provided randomly and blindly as X (Fig 10b). Finally, the participants were asked to respond by pressing either the ‘A’ or ‘B’ key to indicate which of the A or B (***Wood***_***v***_ and ***Jean***_***v***_) matches the provided X. Participants were not informed of *the virtual texture*.

In the experiment, each participant completed 120 ABX test trials (10 pairs × 12 trials), which took approximately 30 minutes to complete. To statistically validate the successful discrimination of two stimuli (a pair of *virtual textures*) at a 95% confidence level, the ABX test required 10 or more correct answers out of 12 trials for each pair. The experiment included ten participants, three females and seven males, with an average age of 28.40 years (SD = 4.93). The ABX test was performed using a touchscreen laptop (Precision 5530 2-in-1, Dell Inc.) with the HAPmini. Each participant was randomly provided ten pairs and the ABX test was conducted 12 times for each pair before moving on to the next pair.

#### 4.2.2 Experimental results

The correctness rate of the ABX test results was 97.3%. This suggests that the participants could distinguish between the five *virtual textures* of the HAPmini. Table 2 lists the correctness rate and completion time required for the ABX test for each pair. The numbers in parentheses indicate the number of subjects who did not pass the ABX test at the 95% confidence level. Two of the ten did not successfully discriminate between ***Wood***_***v***_ and ***Jean***_***v***_. All participants successfully discriminated all other pairs. The average time taken to distinguish each pair showed a tendency similar to that of the correctness rates. It required a short time to distinguish pairs with a high percentage of correct answers, and a long time to distinguish pairs with a low percentage of correct answers.

**Table 2:** Correctness rate and task completion time of ABX test. The number in parentheses indicates the number of participants who failed the ABX test.

**Correctness rate (%)**						**Task completion time (ms)**					
	*Paper*	*Wood*	*Jean*	*Sandpaper*	*Cardboard*		*Paper*	*Wood*	*Jean*	*Sandpaper*	*Cardboard*
*Paper*	-	-	-	-	-	*Paper*	-	-	-	-	-
*Wood*	**96.7 (0)**	-	-	-	-	*Wood*	**3961.3**	-	-	-	-
*Jean*	**95.8 (0)**	**91.7 (2)**	-	-	-	*Jean*	**4652.1**	**6967.4**	-	-	-
*Sandpaper*	**100 (0)**	**96.7 (0)**	**94.2 (0)**	-	-	*Sandpaper*	**3579.9**	**4234.4**	**5224.7**	-	-
*Cardboard*	**100 (0)**	**99.2 (0)**	**100 (0)**	**99.2 (0)**	-	*Cardboard*	**3516.1**	**3329.7**	**3639.1**	**4116.8**	-

***Paper***_***v***_ was rendered with significantly weaker vibration than the other four *virtual textures*. By contrast, ***Sandpaper***_***v***_ and ***Cardboard***_***v***_ were generated through strong vibrations compared with other *virtual textures*. ***Sandpaper***_***v***_ has short and strong vibrations that are repeated to express the sandpaper’s macro-roughness, whereas ***Cardboard***_***v***_ has a long-cycle vibration pattern to express its bumpiness. The distinctive characteristics of the three *virtual textures* made the participants easily distinguish them. However, it appears that the two participants had difficulty distinguishing between ***Wood***_***v***_ and ***Jean***_***v***_, as their vibration amplitudes and patterns were similar to each other when compared to the other vibrations, potentially complicating the discrimination process. However, eight of these successfully discriminated between ***Wood***_***v***_ and ***Jean***_***v***_, whereas the other nine pairs were not successfully discriminated. That is, ten subjects passed 98 tests out of 100 ABX tests (10 participants × 10 pairs) with a 95% confidence level. Overall, the results of the experiment suggested that the participants could differentiate between the five virtual textures generated by HAPmini.

### 4.3. Design Experiment 2–2: Matching test

#### 4.3.1 Experiment design

**Fig 11:**
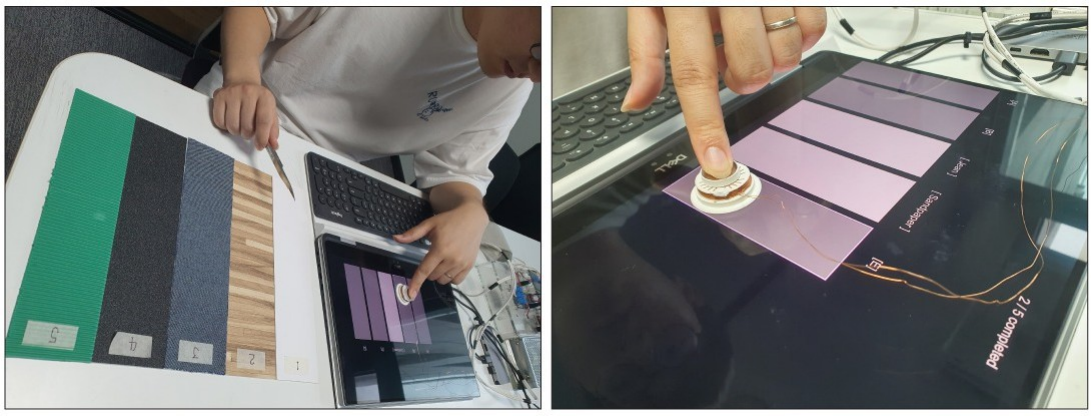


In the matching test, the participants were asked to match each of the five blind-given *virtual textures* (***Paper***_***v***_, ***Wood***_***v***_, ***Jean***_***v***_, ***Sandpaper***_***v***_, *and* ***Cardboard***_***v***_) with the *original textures* (***Paper***_***p***_, ***Wood***_***p***_, ***Jean***_***p***_, ***Sandpaper***_***p***_, *and* ***Cardboard***_***p***_). As shown in Fig 11, five *virtual textures* were blindly provided at positions in a random order. The participants experienced blinded *virtual textures* through HAPmini as well as the *original textures* with their fingers or a cutter knife as much as they wanted. If they were confident about the blinded *virtual texture*, they could answer by pressing the number keys on the keyboard (1: paper, 2: wood, 3: jean, 4: sandpaper, and 5: cardboard). After inputting all answers for the five *virtual textures*, they pressed the ’M’ key to complete the session. The same *virtual texture* was not provided more than once in a single session and duplicate answers were not allowed. Considering the learning effects on the participants, the matching test consisted of eight sessions. Each subject required approximately 15 min to complete the eight matching tests. Ten participants from the ABX test participated in the matching tests.

#### 4.3.2 Experimental results

**Fig 12:**
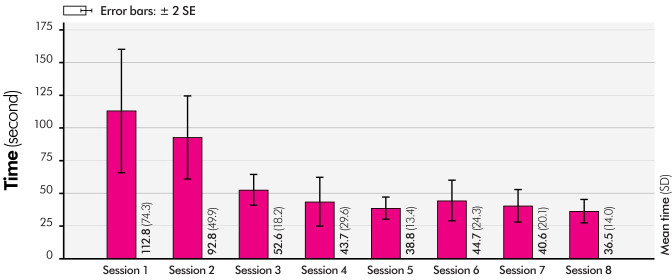


The duration of completion of the matching test was reduced over the sessions (Fig 12), the learning effect disappeared after the third of eight sessions (Bonferroni pairwise comparison, p > 0.05). To minimize the learning effect, the results of the first and second sessions were excluded from analysis. Table 3 shows the correctness rates from the third to the eighth sessions, and the total correctness rate was 93.3%. The correctness rates in Table 3 indicate the success rate of distinguishing each *virtual texture*. From the third to the eighth sessions, a total of 20 errors occurred, and 16 out of 20 errors (80%) occurred between ***Wood***_***v***_ *and* ***Jean***_***v***_. Furthermore, errors occurred twice (two out of 20 errors; 10%) between ***Wood***_***v***_ *and* ***Cardboard***_***v***_, and two times (two out of 20 errors; 10%) between ***Jean***_***v***_ *and* ***Sandpaper***_***v***_. Similar to the results of the ABX test, in the matching test, participants had the most difficulty distinguishing between ***Wood***_***v***_ *and* ***Jean***_***v***_. Nevertheless, the participants could match *the virtual textures* of HAPmini with a high correctness rate (93.3%) to *the original textures*.

**Table 3:** Results of matching test.

**Correctness rate (%)**					
	*Paper*	*Wood*	*Jean*	*Sandpaper*	*Cardboard*
*Paper*	-	-	-	-	-
*Wood*	**100**	-	-	-	-
*Jean*	**100**	**73.3**	-	-	-
*Sandpaper*	**100**	**100**	**96.7**	-	-
*Cardboard*	**100**	**96.7**	**100**	**100**	-

*The virtual textures* of HAPmini were verified using ABX and matching tests. To determine whether HAPmini could successfully deliver various types of textures, five materials with unique characteristics (bumpiness of ***Cardboard***_***p***_, macroroughness of ***Sandpaper***_***p***_, microroughness of ***Paper***_***p***_, and grains of ***Wood***_***p***_ and ***Jean***_***p***_) were selected for testing. In summary, the participants in both experiments could successfully discriminate each pair of *the virtual textures* provided by HAPmini (97.3% correctness rate in the ABX test) and identify where *the virtual textures* originated (93.3% correctness rate in the matching test). By contrast, ***Wood***_***v***_ and ***Jean***_***v***_, whose vibration amplitudes and patterns were similar, were distinguished less successfully from each other in both experiments (91.7% and 73.3% correctness rates in the ABX test and matching test, respectively). Nevertheless, these results show that the participants successfully distinguished between the two at a higher level than simple probabilities (50%). Textures have various properties, such as stickiness, thermal conductivity (warmth or coolness), and roughness, and human beings perceive and recognize textures based on these properties. HAPmini simulates only the mechanical roughness of the texture by generating vibrations and excludes other properties. Although this is not a perfect reproduction of texture, the two experiments demonstrate that HAPmini’s vibrotactile feedback can convey *virtual textures* that are sufficiently distinguishable to users.

## 5. Implications and discussions

### 5.1. Theoretical implications

#### 5.1.1 Enhancing 2-dimensional touch interaction through 1-DOF force feedback

Experiment 1 confirmed that HAPmini can be utilized as an assistive input device for touch interactions, similar to commercial detachable joysticks for touchscreen devices. HAPmini’s force feedback improves the performance of user-pointing tasks and reduces the perceived workload. *The hardware magnetic snap function* can be as effective in touch interaction as *the software magnetic snap function* by restricting the user’s finger movements. Although touch interaction enables users to interact intuitively with graphical elements by freely moving their fingers on a touchscreen, it lacks physical constraints to stably support fingertips moving on a touchscreen. The HAPmini’s hardware joystick can function as an auxiliary input method to aid touch interaction, and the force feedback of HAPmini provides haptic constraints that enhance a user’s task performance by applying force to the user’s finger. In addition, this enables HAPmini to provide 1-DoF force feedback that corresponds to a 2D operation by applying an inward or outward force to the joystick handle.

#### 5.1.2 Texture rendering with vibration along an unregulated axis

In accordance with Lee et al.’s [2] proposition that haptic devices should be designed with low mechanical complexity for practical and commercial purposes, we explored feasible approaches for delivering haptic feedback for various applications using fewer actuators. Because of these efforts, HAPmini features a single solenoid-magnet actuator with coaxial alignment, which can enable joystick input and provide force and tactile feedback for 2-dimensional operation. Previous research has suggested that the direction of vibration has minimal impact on human skin sensation [47]. Based on this foundation, experiments were conducted to investigate whether a coaxial actuator and its vibration along an unregulated axis can accurately render and deliver different types of *virtual textures*. The results showed that HAPmini was able to achieve a high level of accuracy in both the ABX test (97.3% correct answers) and the matching test (93.3% correct answers). Existing vibrotactile feedback devices that generate texture usually use a linear actuators; thus, the direction of vibration is fixed [2,14,20,49]. However, the HAPmini actuator cannot fix or control the direction of vibration; the vibration oscillates in random directions on the 2D plane. However, owing to its coaxial alignment, HAPmini can respond to users’ 2-dimensional touch interaction. HAPmini was an attempt to cope with higher-dimensional manipulation with lower mechanical complexity (i.e., coping with 2-dimensional manipulation with a single actuator). It shows that *virtual textures* could be successfully perceived by the users with the vibration along an unregulated axis.

### 5.2. Practical implications

#### 5.2.1 Adding haptic feedback to conventional interfaces

Many attempts have been made to provide texture information in a touchscreen or VR environment; however, no haptic or tactile device has been successfully utilized in these environments. TeslaTouch [16] has presented promising applications and visions that can utilize electroadhesive tactile feedback in a touchscreen device but requires a touchscreen panel capable of applying a few kilovolts or more. Vibrotactile devices such as HapCube [15] and Waves [14] do not include an input method; therefore, they cannot be directly applied to conventional computing interfaces such as touchscreen devices or VR controllers. TORC [2] and Foldaway haptics [3] provide input means and tactile information simultaneously and can also be implemented in commercial interfaces because of their simple structure. However, as mentioned earlier, TORC provides only vibrotactile feedback, whereas foldaway haptics only generate force feedback. In comparison, HAPmini can simultaneously provide force and vibrotactile feedback, and its size (26 mm × 26 mm × 12 mm) is not significantly different from that of commercial joysticks or other haptic devices. Consequently, it is possible to install HAPmini as a substitute for the physical inputs of conventional interfaces, which is anticipated to enhance the sense of haptics.

#### 5.2.2 Haptic device with low complexity for end users

In this study, we focus on designing a haptic device with low complexity (using only a few actuators and simple structures) that can provide rich haptic feedback. Haptic devices that use solenoid-magnet actuators have been extensively studied. Owing to the fast response speed of the solenoid-magnet actuator, it has been utilized in the study of force and tactile feedback devices. For examples, Pierce et al. [50] used a solenoid-magnet actuator to apply a repulsive force to a user’s fingertip press. Culbertson et al. [14] and Tanabe et al. [49] combined multiple actuators to obtain a multi-DOF pseudo-force feedback. Kim et al. [15] simulated tangential friction and normal compliance by using three perpendicular actuators. However, to the best of our knowledge, the aforementioned studies require multiple actuators to provide haptic feedback that can reflect multi-dimensional movements. Compared with the previous studies, the HAPmini has only one solenoid-magnet actuator and generates force and tactile feedback simultaneously. In addition, the HAPmini can function as a joystick that can recognize two-dimensional movement and, at the same time, provide *the hardware magnetic snap function* and *the virtual textures*. Although not validated, using fewer actuators and a simpler structure would bring users closer to haptic devices. We agree with the assertion of Lee et al. [2]; therefore, it is necessary to develop a haptic device with a low mechanical complexity to make many users experience haptic devices more accessible. Researchers in haptics have developed various types of haptic devices; however, research on the development of haptic devices that can provide rich haptic feedback while reducing mechanical complexity is still in its infancy. HAPmini is designed with a simple structure and fewer actuators than existing devices while being able to deliver various haptic sensations beyond simple buzzing. In addition, it is a 1-DoF haptic device that can correspond to a user’s 2D input. We anticipate that the design of the HAPmini will contribute to the recent trend toward reducing mechanical complexity.

### 5.3. Limitations

#### 5.3.1 Limitation of reproducing texture using only vibration

This study proposed a method of generating *virtual textures* with 1-DoF vibrations. This method uses vibrotactile feedback to stimulate only a portion of the human cutaneous sense, rather than the whole sense. As is well known, four types of mechanoreceptors (i.e., Merkel, Ruffini, Meissner, and Pacinian corpuscles) are involved in the human cutaneous sense [51]. In addition, thermoreceptors that detect temperature are also involved in the perception of texture. Human haptic perception is a result of the synthesis of various receptors. HAPmini, which generates *the virtual textures* using vibrotactile feedback, stimulates only two of the four mechanoreceptors (Meissner and Pacinian corpuscles). The vibrotactile feedback of HAPmini does not stimulate the two mechanoreceptors (Merkel and Ruffini corpuscles) primarily used by blind individuals to recognize Braille. These corpuscles can be stimulated by pin-array-type tactile devices [11,12]. Because *the virtual textures* of HAPmini stimulates only a part of human perception, *the virtual textures* cannot wholly replicate the texture of the actual material. In other words, HAPmini expresses the characteristics of *the original textures* and delivers them to the user by stimulating only two of the four mechanoreceptors involved in the human cutaneous sense (Meissner and Pacinian corpuscles), and therefore only a portion of human haptic perception. To the best of our knowledge, a haptic device that stimulates all human receptors is yet to be proposed.

This study has two limitations. First, in experiment 2–1, an ABX test between the *original textures* and *virtual textures* was not conducted. Thus far, no haptic device has produced a *virtual texture* that is indistinguishable from the *original textures*. Therefore, in this study, the ABX test was conducted only between the *virtual textures*, and the matching test was conducted as a complementary experiment. Second, it has not been demonstrated how closely the *virtual textures* provided by HAPmini resemble *the original textures*. However, the results of the matching tests indicated that users could infer the *original textures* within a limited range (five textures in this study) by perceiving the characteristics of the *virtual textures* represented by HAPmini. In the future, a haptic device capable of stimulating all types of human senses will be developed, and a direct comparative study between original and virtual textures will be conducted.

#### 5.3.2 Wireless design for daily use

Researchers have investigated the potential of haptic devices for everyday use, and we anticipate their incorporation into our daily lives in the near future. Haptic devices using pneumatic actuators require air tanks or air blowers that constantly blow air as well as tubes to connect them [52–54]. Haptic devices that use high voltages typically receive power from power suppliers or voltage generators in laboratory environments [13,16]. Vibrotactile devices are relatively free from power sources; however, they have also been wired to separate power sources for the convenience of development [6,14,55]. For frequent and convenient use of haptic devices, they should be designed wirelessly with low mechanical complexity. Therefore, haptic devices should be able to incorporate power sources, such as small battery packs. Most VR controllers have built-in batteries and operate wirelessly and independently. Although the HAPmini receives power from a separate external power source, it can be installed in commercial VR controllers to share batteries owing to its small size.

## 6. Conclusion

We developed the HAPmini, a device capable of delivering force and tactile feedback to support the users’ touch interaction. HAPmini is an input and output device that can be placed on a touchscreen. The HAPmini functions as a joystick while simultaneously utilizing an electromagnetic force to constrain the direction of movement, as necessary. This enables *the hardware magnetic snap function* by limiting touch movement in the desired target area to support the user’s pointing task on a touchscreen device. In addition, it can successfully generate the specific *virtual textures* of real materials. We discussed the theoretical and practical implications of enhancing the touch interaction. Theoretically, HAPmini enhances the performance of touch interactions and provides *virtual textures* with vibrations along an unregulated axis. Practically, the HAPmini provides force and tactile feedback responding the users’ two-dimensional touch inputs with a signle solenoid-magnet actuator. This design handles two-dimensional operations with only one 1-DoF actuator and is an effort to reduce the mechanical complexity of haptic devices. We anticipate that the HAPmini design efforts (reducing the number of actuators and simplifying the structure) will make it suitable for various conventional interfaces. Furthermore, the incorporation of force and tactile feedback is expected to enhance touch interactions.

## Supporting information

S1 File (ZIP)
